# Preparation and Characterization of PEDOT:PSS/TiO_2_ Micro/Nanofiber-Based Gas Sensors

**DOI:** 10.3390/polym14091780

**Published:** 2022-04-27

**Authors:** Bing-Chiuan Shiu, Yan-Ling Liu, Qian-Yu Yuan, Ching-Wen Lou, Jia-Horng Lin

**Affiliations:** 1 College of Material and Chemical Engineering, Minjiang University, Fuzhou 350108, China; bcshiu@mju.edu.cn; 2 Fujian Key Laboratory of Novel Functional Fibers and Materials, Minjiang University, Fuzhou 350108, China; 3 Innovation Platform of Intelligent and Energy-Saving Textiles, School of Textile Science and Engineering, Tiangong University, Tianjin 300387, China; lyl1504291995@163.com (Y.-L.L.); yuan_qianyu@163.com (Q.-Y.Y.); 4 Department of Medical Research, China Medical University Hospital, China Medical University, Taichung 40402, Taiwan; 5 Advanced Medical Care and Protection Technology Research Center, College of Textile and Clothing, Qingdao University, Qingdao 266071, China; 6 Department of Bioinformatics and Medical Engineering, Asia University, Taichung 413305, Taiwan; 7 Laboratory of Fiber Application and Manufacturing, Department of Fiber and Composite Materials, Feng Chia University, Taichung 40724, Taiwan; 8 School of Chinese Medicine, China Medical University, Taichung 40402, Taiwan

**Keywords:** gas-sensitive nanofiber, micro/nanofiber gas sensor, gas sensitivity, smart wearable device

## Abstract

In this study, we employed electrospinning technology and in situ polymerization to prepare wearable and highly sensitive PVP/PEDOT:PSS/TiO_2_ micro/nanofiber gas sensors. PEDOT, PEDOT:PSS, and TiO_2_ were prepared via in situ polymerization and tested for characteristic peaks using energy-dispersive X-ray spectroscopy (EDS) and Fourier transform infrared spectroscopy (FT-IR), then characterized using a scanning electron microscope (SEM), a four-point probe resistance measurement, and a gas sensor test system. The gas sensitivity was 3.46–12.06% when ethanol with a concentration between 12.5 ppm and 6250 ppm was measured; 625 ppm of ethanol was used in the gas sensitivity measurements for the PEDOT/composite conductive woven fabrics, PVP/PEDOT:PSS nanofiber membranes, and PVP/PEDOT:PSS/TiO_2_ micro/nanofiber gas sensors. The latter exhibited the highest gas sensitivity, which was 5.52% and 2.35% greater than that of the PEDOT/composite conductive woven fabrics and PVP/PEDOT:PSS nanofiber membranes, respectively. In addition, the influence of relative humidity on the performance of the PVP/PEDOT:PSS/TiO_2_ micro/nanofiber gas sensors was examined. The electrical sensitivity decreased with a decrease in ethanol concentration. The gas sensitivity exhibited a linear relationship with relative humidity lower than 75%; however, when the relative humidity was higher than 75%, the gas sensitivity showed a highly non-linear correlation. The test results indicated that the PVP/PEDOT:PSS/TiO_2_ micro/nanofiber gas sensors were flexible and highly sensitive to gas, qualifying them for use as a wearable gas sensor platform at room temperature. The proposed gas sensors demonstrated vital functions and an innovative design for the development of a smart wearable device.

## 1. Introduction

Gas is invisible and the lives of people as well as their livelihoods can be jeopardized by various accidents, including coal mine explosions, sewer biogas incidents, and poisoning incurred by carbon monoxide, the threats of which are unpredictable. Gas sensors are a vital measure to detect the components and concentrations of gas and, as such, effectively control the release and dissemination of toxic gas in living and working spaces. As a result, gas sensors are commonly used in the military, anti-terrorism, industry, home safety, and environmental monitoring; at the same time, wearable electronic textiles are beginning to gain extensive attention via a progressive internet and wearable technology. In other words, wearable textile-based gas sensors are promising products that are worth developing.

Gas sensors have a high sensitivity as well as resilience, durability, and weaveability and can be bonded with textile matrices to form wearable electronic textiles that can be used as convenient detectors for people to examine the transient state of their surroundings to guard their health and prevent toxic gas accidents. Conductive polymers are a recently discovered material [1]. As a thiophene conductive polymer, poly(3,4-ethylenedioxythiophene) (PEDOT) has a high transparency, a stable chemical nature, and good conductivity; these attributes have made PEDOT popular in the widespread research on gas detection [2,3]. As it is more eco-friendly than other conductive polymers such as polyaniline (PANI) and polypyrrole (PPy), PEDOT has gained increasing attention as a tool that can be used to detect the presence of NH_3_, HCL, ethanol, and NO [4]. At present, gas sensors are mostly made of polythiophene [5,6], polyaniline [7], polypyrrole [8], graphene [9,10], titanium dioxide (TiO_2_) [11,12], and zinc oxide (ZnO) [13]. Zhang et al. [14] employed an electrospinning machine equipped with a high-pressure airflow to produce polyvinyl alcohol/poly(3,4-ethylenedioxythiophene):poly(styrene sulfonate) (PVA/PEDOT:PSS) composite ultrafine fibers and characterized the electric properties of the PVA/PEDOT:PSS nanofibers with different diameters. In an attempt to obtain transient response characteristics, Liu et al. [15] prepared PPy-PAN NH_3_ gas sensors using both electrospinning and in situ polymerization. The resulting products were examined for NH_3_ gas detection at a concentration ranging from 250 ppm to 2000 ppm and had a gas sensitivity value ranging between 1.0 and 1.5%. Pang et al. [16] developed a flexible, light, and highly conductive porous graphene network to serve as a humidity sensor.

**Figure 1:**
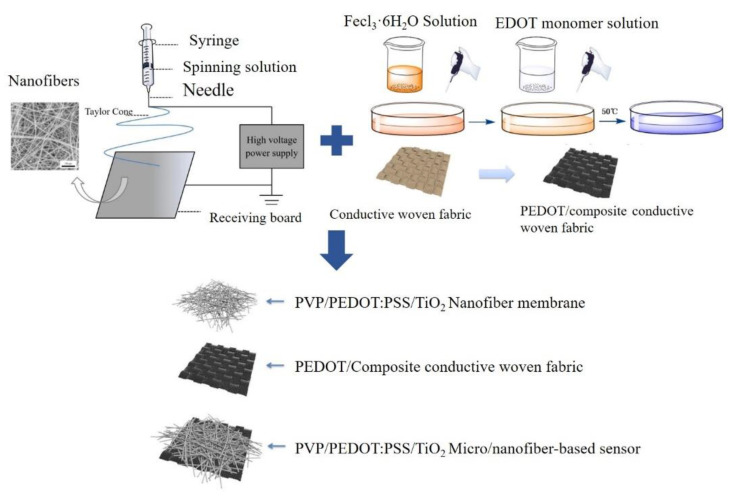
PVP/PEDOT:PSS/TiO_2_ micro/nanofiber gas sensor structure.

With further investigation and development, society has become increasingly fond of TiO_2_ gas-sensitive materials because of their desirable attributes, including a low working environment temperature, an efficient functionality, and ease of production. Based on the findings of the current studies, the performance of TiO_2_ gas-sensitive materials has been improved mainly via atomic doping, heterojunction structure construction, ganic molecular modification, and shape control methods. Notably, the heterojunction structures are very diverse, e.g., PPy/WO_3_ [17], PANI/TiO_2_ [18], and ZnO/TiO_2_/PANI [19], which can concurrently strengthen the gas sensitivity as well as the sensing performance and gas diversity. The main methods used in gas sensor production include electrospinning [20,21], in situ polymerization [22], drip coating and dipping [5], electrostatic self-assembly [9], interfacial polymerization [7], hydrothermal systems [23], nano-level soft lithography [24], and chemical vapor deposition (CVD) [25]. Specifically, electrospun membranes feature a large specific surface area and only require a simple manufacturing process. The electrospun nanofilms have sufficient transmission channels for gas detection; hence, they exhibit a greater gas sensitivity. However, the main chains of the conductive polymers are rigid, which causes lower levels of entanglement and interaction among the polymers. Therefore, it is rather difficult to manufacture PEDOT:PSS nanofibers [26] and their production demands a non-conductive carrier polymer such as polyvinylpyrrolidone (PVP), with a good biocompatibility and film formation. Figure 1 shows the electrospinning assembly.

Fiber- or fabric-based gas sensors are characterized by their ease of processing, low production cost, light weight, and greater sensor efficacy at room temperature. With several advantages, including a low cost along with wearable and portable features, the incorporation of textiles with flexible sensors has found widespread applications [27,28,29]. Fiber- or textile-based gas sensors can be made in different manners to fit fabric products more effectively than conventional solid gas sensors, flexible film sensors, or paper gas sensors [30]. The majority of the studies on textile gas sensors employ electrospinning to produce nanofibers with a large specific surface area; however, there is a relatively small number of studies on textile-type gas sensors. Conductivity is a vital factor for electronic textile-based gas sensors as it directly affects the gas sensitivity. Hence, a rise in conductivity is associated with highly sensitive electronic textile-based gas sensors. Hong et al. used in situ polymerization to prepare PANI-nylon 6 composite fabrics and the products were highly sensitive to NH3 and had a quick reaction time [31]. To produce NH_3_ gas sensors, Wu et al. constructed PANI/PAN with a uniaxial arrangement and coaxial UACNY via electrospinning and in situ polymerization [32]. Yun et al. [33] prepared an electronic textile gas sensor based on reduced graphene oxide (RGO); the resulting gas sensor displayed a 28% response toward 0.45 ppm NO_2_ gas. Lee et al. [34] prepared a graphene-based electronic textile gas sensor that was made of a dopamine–graphene-mixed electronic textile yarn (DGY). The graphene was adhered to the surface of the yarn using dopamine, which allowed the gas sensors to function with a high priority and sensitivity toward NO_2_ gas.

In this study, we used an innovative and convenient in situ polymerization method to coat PEDOT over a conductive woven fabric surface, forming highly conductive and gas-sensitive Cu/Pc-80 composite conductive woven fabrics. The loading level of PEDOT and the gas sensitivity of the products were also tested and analyzed. Finally, the electrospinning technique was used to develop highly sensitive and flexible PVP/PEDOT:PSS/TiO_2_ micro/nanofiber gas sensors. The gas sensitivity and morphology of the resulting gas sensors were then investigated using energy-dispersive X-ray spectroscopy (EDS) analyses, X-ray diffraction (XRD) analyses, and Fourier transform infrared spectroscopy (FT-IR) in order to evaluate their performance.

## 2. Experimental Procedure

### 2.1. Materials

EDOT was purchased from Adamas Reagent Co., Ltd., Shanghai, China. FeCl_3_·6H_2_O, (NH_4_)_2_S_2_O_8_, Na_2_S_2_O_8_, and absolute ethanol (C_2_H_6_O) were purchased from Fengchuan Chemical Reagent Technology Co., Ltd., Tianjin, China. The PVP (Macklin Biochemical Co., Ltd., Shanghai, China) had a molecular weight of 1,300,000 MW. PEDOT:PSS was purchased from Sigma-Aldrich, China, DMSO was obtained from Yuanli Chemical Co., Ltd., Tianjin, China, and TiO_2_ was purchased from Macklin Biochemical Co., Ltd., Shanghai, China.

### 2.2. Instruments

The scanning electron microscope (Phenom pure) was purchased from Phenom Co., Rotterdam, The Netherlands. The electrospinning assembly (JDF05) was acquired from Nayi Instrument Technology Co., Ltd., Changsha, China. The energy-dispersive X-ray spectroscopy analysis was carried out with OCTANE SUPER equipment, purchased from Itax Co., Ltd., USA. The D8 Discover, used for X-ray diffraction, was purchased from Bruker AG, Germany. The Fourier transform infrared spectrophotometer (Nicolet iS50) was purchased from Thermo Fisher Scientific Co., Ltd., Shanghai, China. The gas-sensitive component measurement system (WS-30B) was procured from Weisheng Electronic Technology Co., Ltd., Zhengzhou, China. The vacuum drying oven (DZF-6020) was purchased from Boxun Industrial Co., Ltd., Shanghai, China.

### 2.3. Preparation of PEDOT/Composite Conductive Woven Fabrics

Figure 1 shows the manufacturing process for the PEDOT/composite conductive woven fabrics employing in situ polymerization. First, 0.3 mol/L oxidant (Fecl_3_·6H_2_O) and 0.1 mol/L EDOT monomer were separately dissolved in 10 mL of absolute ethanol. The non-woven fabrics were then placed in dishes, after which the two solutions were separately infused over the fabrics to soak the surface, forming PEDOT/composite conductive woven fabrics. The conductive fabrics were repeatedly washed with deionized water and vitamin C powder to ensure that the oxidants and iron ions were removed.

### 2.4. Preparation of PEDOT:PSS Micro/Nanofiber Gas Sensors

PVP at 8, 9, or 10% was mixed with a water/absolute ethanol (1:3) mixture at 500 r/min for 4 h. A total of 5 g of PEDOT:PSS and DMSO at a 3:1 ratio were individually added to the mixture for another 4 h mixing period and the final mixtures were stored for 12 h. DMSO was added in an attempt to improve the electrical conductivity of the PVP/PEDOT:PSS nanofiber membranes. The PEDOT/composite conductive woven fabrics were then fixed to the roller of the electrospinning assembly. The electrospinning technology was applied to produce PVP/PEDOT:PSS nanofiber membranes with the following parameters: a collection distance of 200 cm, an injection rate of 0.3 mm/L, and an anode high voltage of 25 kV. The collected PVP/PEDOT:PSS nanofiber membranes were dried in a vacuum drying case at 60 °C for 24 h.

### 2.5. Preparation of PEDOT:PSS/TiO_2_ Micro/Nanofiber Gas Sensors

The PVP/PEDOT:PSS/TiO_2_ micro/nanofiber gas sensors were prepared as described above. TiO_2_ was ultrasonically dispersed in water and absolute ethanol in advance. TiO_2_ (0.2 g) was then added to the PVP/PEDOT:PSS (9%) spinning solution systems and stored for 12 h. A certain volume of the electrospinning solutions was infused into a double injector whilst the PEDOT/composite conductive woven fabric was fixed to the roller of the electrospinning assembly. The electrospinning parameters used to produce the PVP/TiO_2_ and PVP/PEDOT:PSS/TiO_2_ nanofiber membranes were as follows: a collection distance of 200 cm, an injection rate of 0.3 mm/L, and an anode high voltage of 25 kV. Finally, the membranes were placed in a vacuum drying oven at 60 °C for 24 h to form the PVP/PEDOT:PSS/TiO_2_ micro/nanofiber gas sensors, as shown in Figure 1.

### 2.6. Testing and Characterization

To evaluate the morphology of the fibers using the SEM, the PEDOT/composite conductive woven fabrics were fixed to the platform of the tester using a conductive glue and coated with a thin layer of gold. A Phenom desktop scanning electron microscope was used to observe the accumulation of PEDOT particles on the conductive woven fabrics at an accelerating voltage of 10 kV. Afterwards, the surface morphology of the PVP/PEDOT:PSS, PVP/PEDOT:PSS/TiO_2_, and PVP/TiO_2_ nanofiber membranes was observed.

Fourier transform infrared spectroscopy (FT-IR; Nicolet iS50) was used to measure the FIR characteristics of the PEDOT/composite conductive woven fabrics, PVP/PEDOT:PSS nanofiber membranes, and PVP/PEDOT:PSS/TiO_2_ nanofiber membranes. The wavelength range was 4000–400 cm^−1^ and the resolution was 0.09 cm^−1^. The energy-dispersive X-ray spectroscopy (EDS) measurements (OCTANE SUPER model) were used to analyze the element content and distribution in the PVP/PEDOT:PSS and PVP/PEDOT:PSS/TiO_2_ nanofiber membranes. The samples were fixed to the platform of the tester in advance for the gold spray treatment. The X-ray diffraction (XRD) measurements (D8 Advance) were used to detect the crystallization phase of the PVP/PEDOT:PSS/TiO_2_ nanofiber membranes. The diffraction target was Cu Kα, the angle range was 20°–80°, the step length was 0.02°, and the scan rate was 0.2 s/step.

**Figure 2:**
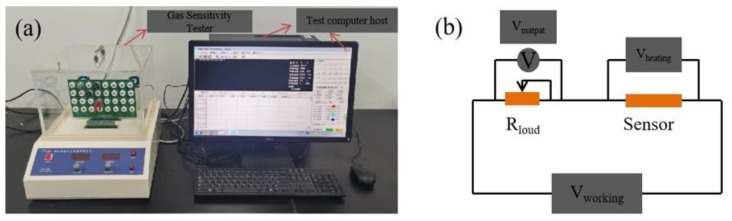
(**a**) Image of gas sensor and (**b**) schematic diagram of test circuit.

A gas sensitivity measurement gas sensor test system (WS-30B) was used to measure the gas sensitivity of the PEDOT/composite conductive woven fabrics, PVP/PEDOT:PSS nanofiber membranes, and PVP/PEDOT:PSS/TiO_2_ nanofiber membranes. Figure 2 shows the gas sensor assembly circuit using ethanol as the test gas. The sensor was incorporated with a voltage of 5 V. Through the suitable load card, the baseline steadily moved between 0 and 1. After two minutes, the corresponding part of the instrument was infused with the specified gas or liquid, after which the detector measured the difference in the electric resistivity. A heater was employed to efficiently evaporate the ethanol and the resulting gas was diffused into the whole test case via a blowing air set. The test was conducted at room temperature and air served as the carrier gas, thereby simulating a common sensor environment. The materials were evaluated for gas sensitivity and its relation to the relative humidity. The electric resistivity (S) was the comparison of the gas sensor before and after the test, and could be written as follows:

S = (Rg − Ra)/Ra × 100%(1)

where Ra is the electric resistivity of the gas sensor materials in the air and Rg is the electric resistivity of the gas sensor materials in the test gas.

## 3. Results and Discussion

### 3.1. Surface Morphology of PEDOT/Composite Conductive Woven Fabrics

**Figure 3:**
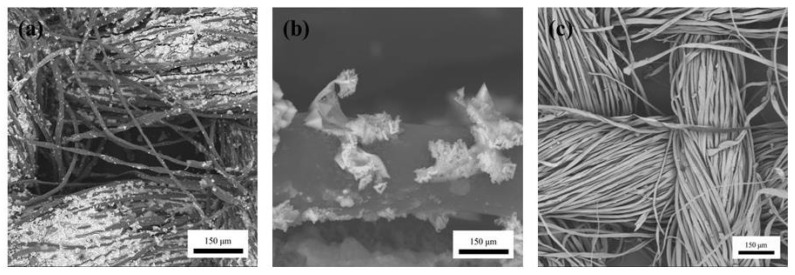
Surface morphology of (**a**,**b**) PEDOT/composite conductive woven fabric and (**c**) Cu/Pc-80 conductive woven fabric.

Figure 3a shows the morphology of the optimal PEDOT/composite conductive woven fabrics; a magnified image is shown in Figure 3b. Figure 3c demonstrates the morphology of the Cu/Pc-80 conductive woven fabrics (i.e., the control group). Compared with the control group, the PEDOT/composite conductive woven fabrics were unevenly coated with the polymer particles. From a macro perspective, the white surface of the fabrics turned dark blue, which suggested that PEDOT had been successfully loaded over the Cu/Pc-80 woven fabrics to form the PEDOT/composite conductive woven fabrics.

### 3.2. FT-IR Analysis of PEDOT/Conductive Woven Fabrics

**Figure 4:**
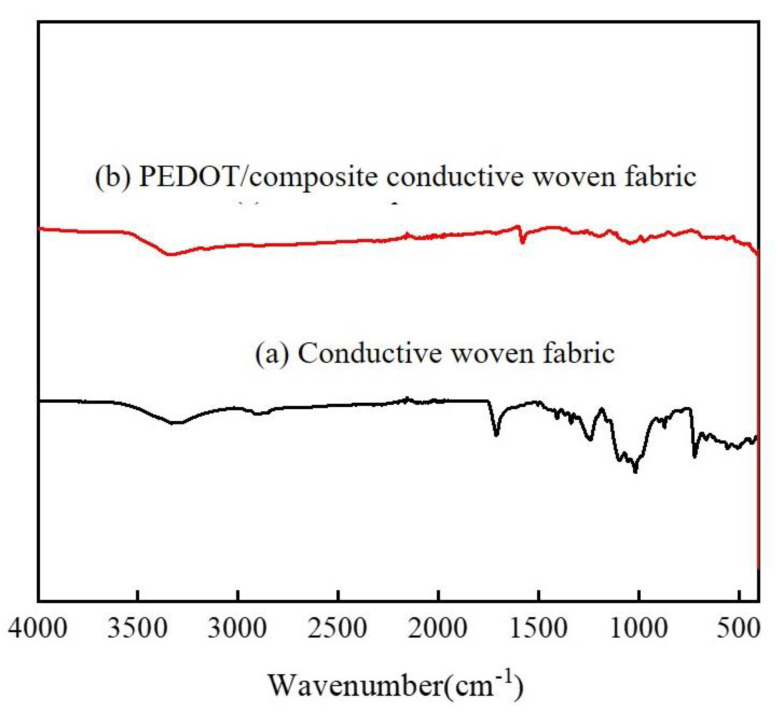
FT−IR spectra of (**a**) Cu/Pc−80 conductive woven fabrics and (**b**) PEDOT/composite conductive woven fabrics.

Figure 4 shows the FT-IR spectra of the Cu/Pc-80 conductive woven fabrics and the PEDOT/composite conductive woven fabrics at 500–4000 cm^−1^. Both groups showed similar FT-IR bands; this once again validated that PEDOT had been successfully polymerized over the surface of the Cu/Pc-80 conductive woven fabrics, which in turn changed the spectra. Hence, in the PEDOT/composite conductive woven fabrics, the characteristic peaks of PEDOT were present at 900–1520 cm^−1^, including stretch peaks at 1576 and 1320 cm^−1^ for C=C and C–C, respectively; stretch peaks at 1210, 1125, and 1051 cm^−1^ for C–O–C in ethylenedioxy; and peaks at 967, 822, and 682 cm^−1^ for C–S in the thiophene ring [4].

### 3.3. Gas Sensitivity of PEDOT/Composite Conductive Woven Fabrics

**Figure 5:**
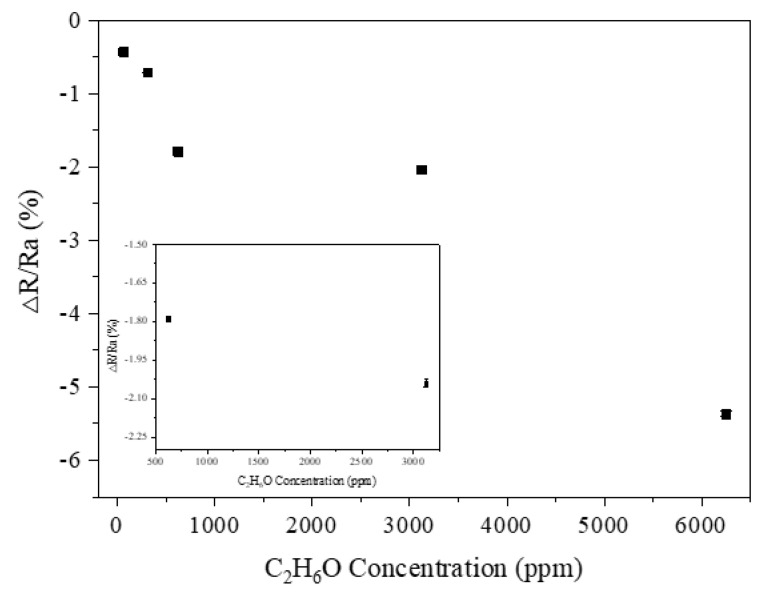
Response curve of PEDOT/composite woven fabrics in relation to ethanol concentration.

Five different concentrations (62.5, 312.5, 625, 3125, and 6250 ppm) of ethanol were individually heated until they reached full volatility to obtain the ethanol gas. When exposed to the ethanol gas, the PEDOT/composite conductive woven fabrics reacted within 180 s and showed differences in electric resistivity (kΩ). Figure 5 demonstrates the gas sensitivity of the PEDOT/composite conductive woven fabrics in relation to the ethanol concentration. The test results showed that the incorporation of PEDOT provided the fabrics with gas sensitivities of 0.43% for an ethanol concentration of 62.5 ppm and 5.37% for a concentration of 6250 ppm, respectively. Therefore, the gas sensitivity of the PEDOT/composite conductive woven fabrics was directly proportional to the ethanol concentration. According to Figure 3a,c, unlike the Cu/Pc-80 conductive woven fabrics, the PEDOT/composite conductive woven fabrics were coated with a considerable amount of PEDOT. Due to its intrinsic conductivity, PEDOT enabled the PEDOT/composite conductive woven fabrics to detect the gas by providing the test gas with enough conductive transmission tunnels over the Cu/Pc-80 conductive woven fabrics.

### 3.4. Morphology Characterization of Gas-Sensing Membranes

**Figure 6:**
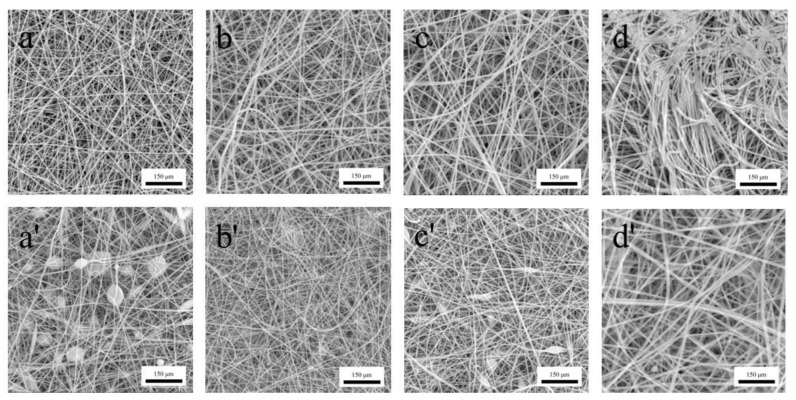
Morphology and structure of the nanofibers of (**a**–**c**) pure PVP membranes and (**a’**–**c’**) PEDOT:PSS nanofiber membranes with a PVP content of 8, 9, and 10%. Morphology of (**d**) pure PVP/TiO_2_ membranes and (**d’**) PVP/PEDOT:PSS/TiO_2_ membranes at a specified PVP content of 9%.

The morphology of the nanofibers was observed using a scanning electron microscope. Figure 6a–c,a’–c’ show the morphology of the pure nanofiber membranes and PEDOT:PSS nanofiber membranes in relation to the PVP content (8, 9, and 10%), respectively. When the PVP content was 8 and 10%, the resulting PVP/PEDOT:PSS nanofiber membranes generated a considerable number of bead-like nanofibers. In contrast, a PVP content of 9% provided the PVP/PEDOT:PSS nanofiber membranes with a morphology that did not contain bead-like nanofibers. A lower PVP content resulted in the electrospinning solution comprising a greater proportion of the solvent. The solvent could not evaporate completely, resulting in the considerable presence of bead-like nanofibers. Moreover, the nanofibers demonstrated a diameter and morphology that were directly dependent on the electrospinning concentration. An excessively low or excessively high concentration caused a high force variation between the surface tension of the solution and the electrospinning force, which was associated with uneven splitting [31]. As shown in Figure 6d,d’, at a specified PVP concentration of 9%, the PVP/TiO_2_ nanofiber membranes exhibited nanofibers with larger diameters and an irregular alignment whereas the PVP/PEDOT:PSS/TiO_2_ nanofiber membranes exhibited nanofibers with smaller diameters and a sleek surface.

### 3.5. EDS Analysis of Gas-Sensing Membranes

**Figure 7:**
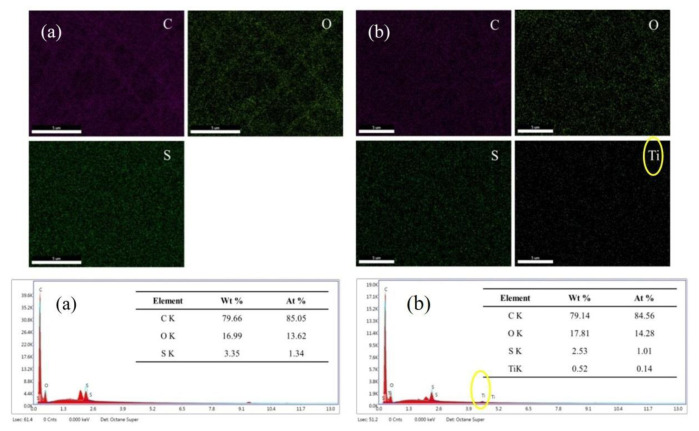
EDS analysis of (**a**) PVP/PEDOT:PSS and (**b**) PVP/PEDOT:PSS/TiO_2_ nanofiber membranes.

An EDS analysis was conducted to characterize the elements on the surface of the PVP/PEDOT:PSS nanofiber membranes and PVP/PEDOT:PSS/TiO_2_ nanofiber membranes; the results are displayed in Figure 7a,b. As shown in Figure 7a, the analysis revealed large amounts of C, O, and S elements, which indicated that a considerable amount of PEDOT:PSS was electrospun into the nanofiber membranes. Moreover, as shown in Figure 7b, the PVP/PEDOT:PSS/TiO_2_ nanofiber membranes contained Ti in addition to C, O, and S. The presence of a weight ratio of 0.52%, representative of Ti derived from TiO_2_, indicated that the TiO_2_ powder successfully bonded with the PVP/PEDOT:PSS/TiO_2_ nanofiber membranes. In addition, the EDS analysis substantiated the presence of TiO_2_, as evidenced by the characteristic peaks of Ti indicated by yellow circles in Figure 7b.

### 3.6. XRD Analysis of Gas-Sensing Membranes

**Figure 8:**
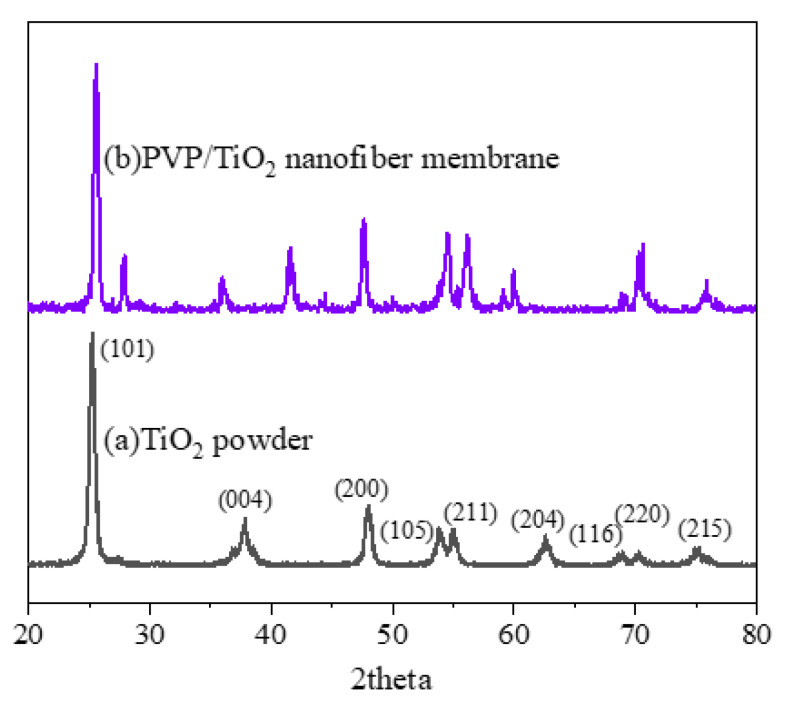
XRD patterns of (**a**) pure TiO_2_ powder and (**b**) PVP/TiO_2_ nanofiber membranes.

For the PVP/PEDOT:PSS/TiO_2_ nanofiber membranes, the 0.2 g of TiO_2_ added was relatively small compared with the PEDOT:PSS content, which may have affected the XRD pattern of the TiO_2_. Therefore, XRD measurements were collected in order to examine whether TiO_2_ was present in the PVP/PEDOT:PSS/TiO_2_ nanofiber membranes. Figure 8a,b shows the XRD patterns of the TiO_2_ powder and PEDOT:PSS/TiO_2_ nanofiber membranes. The TiO_2_ nanocrystals produced peaks at 2 θ with corresponding crystal phases at 25.27° (101), 37.85° (004), 48.07° (200), 53.79° (105), 55.03° (211), 62.59° (204), 68.91° (116), 70.31° (220), and 75.19° (215) [35,36,37]. Meanwhile, the diffraction peaks of the PEDOT:PSS/TiO_2_ nanofiber membranes resembled those of the TiO_2_ nanopowder, with peaks at 29.45° (101), 39.39° (004), 43.22° (200), 47.58° (105), 48.63° (211), 50.96° (204), 56.4° (116), 57.62° (220), and 60.92° (215). The PVP/PEDOT:PSS/TiO_2_ nanofiber membranes were prepared with PVP/TiO_2_ mixtures composed of (3:1) PEDOT:PSS and DMSO solutions followed by the electrospinning process. There were peaks characteristic of TiO_2_ in the XRD pattern of the PVP/TiO_2_ nanofiber membrane, which proved that the PVP/PEDOT:PSS/TiO_2_ nanofiber membranes contained TiO_2_ nanopowder.

### 3.7. FT-IR Analysis of Gas-Sensing Membranes

**Figure 9:**
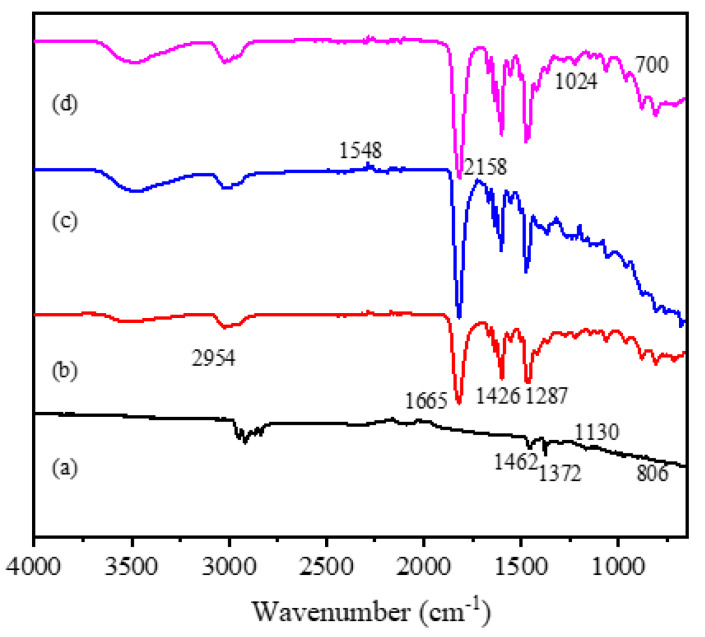
FT-IR diagram of (**a**) PEDOT:PSS solution, (**b**) pure PVP nanofiber membranes, (**c**) PVP/PEDOT:PSS nanofiber membranes, and (**d**) PVP/PEDOT:PSS/TiO_2_ nanofiber membranes.

Figure 9 shows the FT-IR spectra of both the PVP/PEDOT:PSS and PVP/PEDOT:PSS/TiO_2_ micro/nanofiber gas sensors, which further substantiated the presence of PEDOT:PSS and TiO_2_ in the electrospun membranes. Figure 9a–d presents the FT-IR spectra for the PEDOT:PSS solution, pure PVP nanofiber membranes, PVP/PEDOT:PSS nanofiber membranes, and PVP/PEDOT:PSS/TiO_2_ nanofiber membranes. As shown in Figure 9a, the PEDOT:PSS solution showed peaks at 2912 cm^−1^ characteristic of C=O stretching [38]; peaks at 1642 cm^−1^ corresponded with the stretching of the C=C pendant phenyl group and EDOT quinoid and peaks at 1372 cm^−1^ corresponded with the C–C stretching of the thiophene ring. Moreover, peaks at 1130 cm^−1^ and 806 cm^−1^ corresponding with the stretching of the S−OH molecules and SO_3_H groups in PSS, respectively, were also present [39].

As shown in Figure 9b, the pure PVP nanofiber membrane exhibited a C–H stretching vibration peak at 2954 cm^−1^ [38] and a C = O stretching vibration absorption peak at 1665 cm^−1^, which were characteristic of PVP. Meanwhile, the peaks at 1462 cm^−1^ and 1287 cm^−1^ were attributed to C–H flexural vibration absorption and C–N stretching vibration absorption, respectively. As shown in Figure 9c for the PVP/PEDOT:PSS nanofiber membranes, the peaks at 2158 cm^−1^ were ascribed to C–O–C absorption whereas the peaks at 1548 cm^−1^ were attributed to C=C absorption caused by vibration in the thiophene ring. Finally, in Figure 9d, the peaks at 1024 cm^−1^ exhibited by the PVP/PEDOT:PSS/TiO_2_ nanofiber membranes indicated the stretching of the metal oxide bond (Ti–O–Ti) [39]. The presence of peaks characteristic of TiO_2_ indicated that TiO_2_ was successfully incorporated into the PVP/PEDOT:PSS/TiO_2_ micro/nanofiber gas sensors.

### 3.8. Gas Sensitivity Response

**Figure 10:**
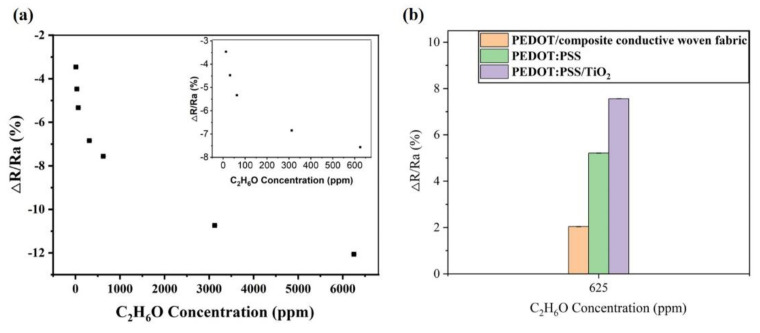
(**a**) The response curve of PVP/PEDOT:PSS/TiO_2_ micro/nanofiber gas sensors exposed to different concentrations of ethanol (62.5 ppm-6250 ppm). (**b**) Comparative response curve of different sensors with a specified ethanol concentration of 625 ppm.

Figure 10a shows the gas sensitivity response–gas concentration (12.5 ppm–6250 ppm) curve of the PVP/PEDOT:PSS/TiO_2_ micro/nanofiber gas sensor. It was produced based on the principle that the conductivity of the porous conductor (TiO_2_) changed with a change in the oxygen content of the exhaust gas; this is also called a resistive oxygen sensor. A gas-sensitive film made of these metal oxides is an impedance device and ions can be exchanged between the gas molecules and the sensitive film [40]; a reduction reaction then occurs, resulting in a change in the resistance of the sensitive film and the dependence of the sample on the gas concentration can be investigated. The gas sensitivity response was 12.06% for a concentration of 6250 ppm and 3.46% for a concentration of 12.5 ppm; these results showed that, following a rise in ethanol concentration, the gas sensors exhibited an improved response due to a greater gas sensitivity. The PEDOT:PSS micro/nanofiber gas sensors proposed by Dan et al. exhibited a gas sensitivity of 0.06% for 76 ppm methanol vapors, 0.14% for 110 ppm ethanol vapors, and 0.5% for 120 ppm acetone vapors [41]. Figure 10b shows the gas sensitivity to 625 ppm ethanol, which was 7.56%, 2.04%, and 5.21% for the PVP/PEDOT:PSS/TiO_2_ micro/nanofiber gas sensors, PEDOT/composite conductive woven fabrics, and PEDOT:PSS micro/nanofiber gas sensors, respectively. The PVP/PEDOT:PSS/TiO_2_ micro/nanofiber gas sensors clearly outperformed the other two groups in terms of the gas sensitivity response, which was ascribed to the synergistic effect and compensation behavior between the PEDOT:PSS and TiO_2_. In addition, regardless of the ethanol concentration, the PEDOT/composite conductive woven fabrics (i.e., the components) had an influence on the continuous gas sensitivity response–recovery curves of the resulting PVP/PEDOT:PSS/TiO_2_ micro/nanofiber gas sensors. 

The high sensitivity of the PVP/PEDOT:PSS/TiO_2_ micro/nanofiber gas sensors could be correlated with many influential factors such as the gas sensitivity and the nanostructure of the PEDOT:PSS nanofiber membranes as well as the potential CP doping effect of TiO_2_. Furthermore, PVP is insulating, which excluded the possibility of transfer incurred by the mutual reaction between the charge and steam. On the other hand, the polymer exerted gas sensitivity as a result of the expansion process as well as the interaction between the polymers and the volatile organic compounds. Notably, the PVP/PEDOT:PSS/TiO_2_ micro/nanofiber gas sensors containing 0.2 g of TiO_2_ exhibited a maximal response toward the ethanol. As PEDOT:PSS and TiO_2_ can build a heterostructure (i.e., the P–N structure), the CP electronic structure was transferred and the gas sensitivity was strengthened accordingly. The interaction with the gas after adsorption could easily adjust the conductivity of the heterojunction [42,43].

**Figure 11:**
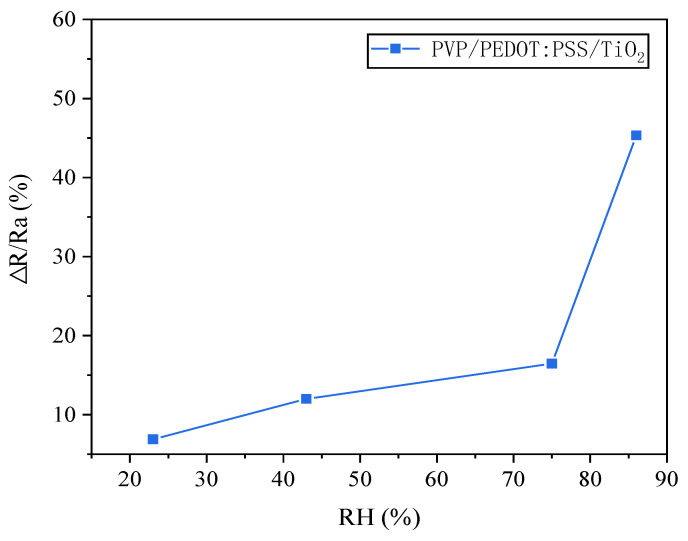
The influence of relative humidity (11–86%) on the gas sensitivity response of the sensors.

Exposure to colorless ammonia vapor can result in the failure of human organs or even death; the higher the humidity, the easier it is for ammonia vapor to enter the body [44]. Additionally, PEDOT:PSS also exhibits a greater gas sensitivity response with an increase in relative humidity [45]; therefore, the gas sensitivity response of the PVP/PEDOT:PSS/TiO_2_ micro/nanofiber gas sensors in relation to the relative humidity was examined in this study, as shown in Figure 11. Through the incorporation of a saturated salt solution composed of LiCl, MgCl_2_, NaCl, and KCl in glass containers, humidities of 11, 23, 43, 75, and 86% were acquired [46], after which the sensors were placed into the glass containers to examine the gas sensitivity response. The PVP/PEDOT:PSS/TiO_2_ micro/nanofiber gas sensors demonstrated a high response capability in relation to the relative humidity (RH). The maximal gas sensitivity response occurred at an RH of 75%, demonstrating a highly non-linear response due to the presence of a water meniscus over the PVP/PEDOT:PSS/TiO_2_ micro/nanofiber gas sensors [47]. When the RH was lower than 75%, the PVP/PEDOT:PSS/TiO_2_ micro/nanofiber gas sensors functioned as a humidity sensor.

## 4. Conclusions

In this study, wearable, highly sensitive, reversible, recyclable, and ultrasoft gas sensors were produced from PEDOT/composite conductive woven fabrics and PVP/PEDOT:PSS/TiO_2_ micro/nanofiber gas sensors using in situ polymerization. The serial performances of the products were evaluated and the results could be summarized as follows. The PEDOT/composite conductive woven fabrics had a basic sensing function and exhibited a sensitivity of 0.43% and 5.37% when the ethanol concentration was 62.5 ppm and 6250 ppm, respectively. The PVP/PEDOT:PSS/TiO_2_ micro/nanofiber gas sensors were successfully produced using electrospinning technology and showed a higher gas sensitivity than that of the PEDOT/composite conductive woven fabrics and the PVP/PEDOT:PSS micro/nanofiber gas sensors. The resulting ethanol gas sensitivity response was 3.46% for 12.5 ppm ethanol and 12.06% for 6250 ppm ethanol. At 625 ppm, the gas sensitivity of the PVP/PEDOT:PSS/TiO_2_ micro/nanofiber gas sensors was 5.52% and 2.35% higher than that of the PEDOT/composite conductive woven fabrics and the PVP/PEDOT:PSS micro/nanofiber gas sensors, respectively. Moreover, the gas sensitivity response of the PVP/PEDOT:PSS/TiO_2_ micro/nanofiber exhibited a linear relationship with the humidity when the humidity was lower than 75%; however, the opposite was the case for a humidity higher than 75%. The improved production techniques for PVP/PEDOT:PSS/TiO_2_ micro/nanofiber gas sensors proposed here are valuable as they provide a basis for the development of smart clothing from an innovative perspective. Many gas sensors based on conductive fabrics are not as effective as metal-based metal oxide semiconductor sensors [48]; however, one advantage is that they can be implemented in wearables. High-precision fiber-based gas sensors produce irreversible changes such as discoloration once the target gas is detected [49]. Studies on conductive textile-based gas sensors need to show repeatability; however, to improve their performance, the conductive properties of conductive textiles are required. Thus, improvements can be made to effectively and significantly improve their gas-sensing performance.

## Data Availability

Not applicable.
